# Lipid and metabolic alteration involvement in physiotherapy for chronic nonspecific low back pain

**DOI:** 10.1186/s12944-022-01737-4

**Published:** 2022-11-25

**Authors:** Zhou Zhang, Chanjuan Zhang, Yuelong Li, Chuhuai Wang, Qiuhua Yu

**Affiliations:** Department of Rehabilitation Medicine, The First Affiliated Hospital, Sun Yat-sen University, 510275 Guangzhou, P. R. China

**Keywords:** Chronic nonspecific low back pain, Lipid, Metabolite, Manual therapy, Therapeutic exercise

## Abstract

**Background:**

Chronic nonspecific low back pain (cNLBP) is a common health problem worldwide, affecting 65–80% of the population and greatly affecting people’s quality of life and productivity. It also causes huge economic losses. Manual therapy (MT) and therapeutic exercise (TE) are effective treatment options for cNLBP physiotherapy-based treatment. However, the underlying mechanisms that promote cNLBP amelioration by MT or TE are incompletely understood.

**Methods:**

Seventeen recruited subjects were randomly divided into an MT group and a TE group. Subjects in the MT group performed muscular relaxation, myofascial release, and mobilization for 20 min during each treatment session. The treatment lasted for a total of six sessions, once every two days. Subjects in the TE group completed motor control and core stability exercises for 30 min during each treatment session. The motor control exercise included stretching of the trunk and extremity muscles through trunk and hip rotation and flexion training. Stabilization exercises consisted of the (1) bridge exercise, (2) single-leg-lift bridge exercise, (3) side bridge exercise, (4) two-point bird-dog position with an elevated contralateral leg and arm, (5) bear crawl exercise, and (6) dead bug exercise. The treatment lasted for a total of six sessions, with one session every two days. Serum samples were collected from subjects before and after physiotherapy-based treatment for lipidomic and metabolomic measurements.

**Results:**

Through lipidomic analysis, we found that the phosphatidylcholine/phosphatidylethanolamine (PC/PE) ratio decreased and the sphingomyelin/ceramide (SM/Cer) ratio increased in cNLBP patients after MT or TE treatment. In addition, eight metabolites enriched in pyrimidine and purine differed significantly in cNLBP patients who received MT treatment. A total of nine metabolites enriched in pyrimidine, tyrosine, and galactose pathways differed significantly in cNLBP patients after TE treatment during metabolomics analysis.

**Conclusion:**

Our study was the first to elucidate the alterations in the lipidomics and metabolomics of cNLBP physiotherapy-based treatment and can expand our knowledge of cNLBP physiotherapy-based treatment.

## Introduction

Chronic nonspecific low back pain (cNLBP) is not caused by recognizable pathology; it lasts for more than three months and occurs between the lower rib and the inferior gluteal fold [1]. It is estimated that approximately four out of five people have lower back pain at some time during their lives, and it greatly affects their quality of life, productivity, and ability to work [2].

cNLBP can be caused by many factors, such as lumbar strain, nerve irritation, and bony encroachment. However, the etiology of cNLBP is typically unknown and poorly understood [3]. Medical treatments and physiotherapy are recommended to treat and resolve issues associated with cNLBP [3]. Therapeutic exercise and manual therapy have a lower risk of increasing future back injuries or work absence and are more effective treatment options for chronic pain than medication or surgery, and they can be performed at rehabilitation clinics [4–6]. Exercise therapy is a widely used strategy to cope with low back pain that includes a heterogeneous group of interventions ranging from aerobic exercise or general physical fitness to muscle strengthening and various types of flexibility and stretching exercises [7]. Manual therapy is another effective method to deal with low back pain, in which hands are used to apply a force with a therapeutic intent, including massage, joint mobilization/manipulation, myofascial release, nerve manipulation, strain/counter strain, and acupressure [8]. However, the reasons therapeutic exercise and manual therapy ameliorate cNLBP are still unknown.

With the development of lipidomics and metabolomics, many studies have indicated that lipid or metabolite alterations are associated with chronic pain [9, 10]. Lipids, as primary metabolites, are not only structural components of membranes but can also be used as signaling molecules to regulate many physiological activities. For example, fatty acid (FA) chains can be saturated (SFA), monounsaturated (MUFA), or polyunsaturated (PUFA), and the ratio of saturated to unsaturated FAs participates in the regulation of longevity [11]. Phosphatidylcholine (PC) phosphatidylethanolamine (PE) is abundant in membranes. In mammals, cellular PC/PE molar ratios that are out of balance and increase or decrease abnormally can cause diseases [12]. For example, a reduced PC/PE ratio can protect mice against atherosclerosis [13]. Decreasing the PC/PE molar ratio can change the intracellular energy supply by activating the electron transport chain and mitochondrial respiration [14]. Lysophosphatidylcholine (LPC) 16:0 correlated with pain outcomes in a cohort of patients with osteoarthritis [15]. Apart from phospholipids, studies have shown that sphingolipid metabolism also contributes to chronic pain. Increased ceramide and sphingosine-1-phosphate (S1P) are involved in the progression of chronic pain in the nervous system [16]. Previous studies reported that metabolites were also associated with pain. Patients with neuropathic pain showed elevated choline-containing compounds in response to myoinositol [tCho/mI] under magnetic resonance spectroscopy [17]. Flavonoids are the most common secondary plant metabolites used as tranquilizers in folkloric medicine and have been claimed to reduce neuropathic pain [18]. Patients with chest pain and high plasma levels of deoxyuridine, homoserine, and methionine had an increased risk of myocardial infarction [19]. Despite the evidence presented above that pain is associated with specific lipids and metabolites, no studies have shown that MT and TE can relieve cNLBP by altering lipids and metabolites.

In this article, we compared the lipidomics and metabolomics of patients with cNLBP before and after treatment to explore differences in lipids and metabolites correlated with cNLBP physiotherapy-based treatment. The newly found data will expand our knowledge of cNLBP physiotherapy-based treatment.

## Material and methods

### Participants

Patients with cNLBP were recruited through advertising. The inclusion criteria were as follows: (1) patients aged between 18 years and 65 years [20]; (2) patients with pain in the area between the lower rib and the inferior gluteal fold; (3) patients with persistent pain > 3 months or intermittent pain > 6 months and having been clinically diagnosed as having cNLBP by two licensed medical doctors in accordance with the diagnostic guidelines published by the American College of Physicians and the American Pain Society [21, 22]; (4) patients with a minimum score of 2 on the Visual Analog Scale (VAS) in the previous week [23]; (5) patients who were right-hand dominant, with no neurological diseases (e.g., traumatic brain injury, or epilepsy), or intracranial lesions; and (6) patients who did not receive pain treatment within the past 3 months.

The exclusion criteria were as follows: (1) patients with radiating pain, menstrual pain, recent/current pregnancy, or postpartum low back pain; (2) patients who suffered known inflammatory disease of the spine, vertebral fracture, severe osteoporosis, autoinflammatory arthritis, and cancer or had significant unexplained weight loss; (3) patients who had cardio-cerebrovascular disease or endocrine disorders; (4) patients with mental illness requiring immediate pharmacotherapy; (5) patients who showed an unwillingness to sign research consent and unwillingness or inability to follow the research protocol; and (6) patients with current alcohol or drug dependence.

All participants were assessed for pain intensity using the visual analog scale (VAS), and serum samples for LC‒MS measurements were collected before and after treatment. The First Affiliated Hospital of Sun Yat-sen University approved the ethical approval document of the study (no. [2019] 408). The recruited subjects signed informed consent forms prior to the experiment.

### Therapy of subjects

Seventeen recruited subjects were randomly divided into the MT group and the TE group. Patients in the MT group received manual therapy, and patients in the TE group received therapeutic exercise. Subjects in the manual therapy group were involved in muscular relaxation, myofascial release, and mobilization for 20 min during each session. The treatment lasted for a total of six sessions, once every two days. Subjects in the therapeutic exercise group completed motor control exercise and core stability exercise for 30 min during each session. The motor control exercises included stretching of the trunk and extremity muscles, trunk and hip rotation, and flexion training. Stabilization exercises consisted of the (1) bridge exercise, (2) single-leg-lift bridge exercise, (3) side bridge exercise, (4) two-point bird-dog position elevated contralateral leg and arm, (6) bear crawl exercise, and (7) dead bug exercise. The treatment lasted for a total of six sessions, once every two days.

### Lipidomic analysis

Lipid samples were prepared as described by Xuan et al. with some modifications [24]. Briefly, venous blood was collected in heparinization tubes and then centrifuged for 15 min at 2000 g at 4 °C to collect serum. A total of 200 µL serum samples with lipid standards were mixed with 400 µL tert-butyl methyl ether (MTBE) and 80 µL methanol and then vortexed for 30 s. Next, the samples were centrifuged, after which the upper phases were collected, transferred into new tubes, and dried by vacuum evaporation. Samples were reconstituted with 100 µL of methylene chloride:methanol (1:1, *v/v*).

Lipid analysis was carried out with a Shimadzu LC-30 A (Shimadzu, Kyoto, Japan) coupled with a mass spectrometer (QTRAP 6500, AB SCIEX, Framingham, MA, USA). The chromatographic parameters were as follows: chromatographic column: ACQUITY UPLC® BEH C18 column (2.1 × 100 mm, 1.7 μm, Waters, Milford, MA, USA), volume of injection: 5 µl, flow rate: 0.26 mL/min, oven temperature: 55 °C. The mobile phase included reagent A (acetonitrile:ultrapure water = 60:40, *v/v*, with 10 mM ammonium acetate) and reagent B (isopropanol:acetonitrile = 90:10, *v/v*, with 10 mM ammonium acetate). A binary gradient was set as follows: 0–1.5 min, mobile phase including 68% reagent A and 32% reagent B; 1.5–15.5 min, mobile phase including 15% reagent A and 85% reagent B; 15.5–15.6 min, mobile phase including 3% reagent A and 97% reagent B; 15.6–18 min, mobile phase including 3% reagent A and 97% reagent B; 18–18.1 min, mobile phase including 68% reagent A and 32% reagent B; 18.1–20 min, mobile phase including 68% reagent A and 32% reagent B. Electrospray ionization (QTRAP 6500, AB SCIEX, Framingham, MA, USA) was used with the following parameters: ion source voltage was − 4500 or 5500 V, ion source temperature was 600 °C, curtain gas was 20 psi, atomizing gas was 60 psi, and auxiliary gas was 60 psi. Scanning was performed through multiple reaction monitoring (MRM). Samples under test conditions were mixed and used as QC samples for LC‒MS analysis every third sample to correct deviations caused by instrumental drift and evaluate the quality of data.

### Metabolomic measurement

Metabolomic samples were prepared as described by Wang et al. [25]. Briefly, venous blood was collected in heparinization tubes and then centrifuged at 8000 g at 4 °C to collect serum. A total of 100 µL of serum sample was mixed with 400 µL of solution (methanol:acetonitrile:ultrapure water = 2:2:1, *v/v/v*) and then sonicated for 10 min in a 4 °C water bath. Next, the samples were incubated for one hour at − 20 °C and then centrifuged. The supernatant was collected and evaporated by vacuum evaporation. Each sample was resuspended in solution (acetonitrile:ultrapure water, 1:1, *v/v*).

Metabolomic analysis was carried out with a Shimadzu LC-30 A (Shimadzu, Kyoto, Japan) coupled with a mass spectrometer (QTRAP 4500, AB SCIEX, Framingham, MA, USA). The chromatographic parameters were set as follows: chromatographic column: UPLC BEH Amide column (2.1 × 100 mm, 1.7 μm, Waters, Milford, MA, USA), volume of injection: 5 µl, flow rate: 0.3 mL/min, oven temperature: 55 °C. The mobile phase included reagent A (100% ultrapure water with 0.025 M ammonium hydroxide and 0.025 M ammonium acetate) and reagent B (100% acetonitrile). A binary gradient was set as follows: 0–1 min, mobile phase including of 15% reagent A and 85% reagent B; 1–12 min, mobile phase including of 35% reagent A and 65% reagent B; 12–12.1 min, mobile phase including of 60% reagent A and 40% reagent B; 12.1–15 min, mobile phase including of 60% reagent A and 40% reagent B; 15–15.1 min, mobile phase including of 15% reagent A and 85% reagent B; 15.1–20 min, mobile phase including of 15% reagent A and 85% reagent B. An electrospray ionization (QTRAP 4500, AB SCIEX, Framingham, MA, USA) was used with parameters as follows: ion source voltage was − 4500 or 5500 V, ion source temperature was 600 °C, the ion source voltage was − 4500 or 5500 V, curtain gas was 20 psi, atomizing gas was 60 psi, and auxiliary gas was 60 psi. Scanning was performed via multiple reaction monitoring (MRM). Samples under test conditions were mixed and used as QC samples for LC‒MS analysis every third sample to correct deviations caused by instrumental drift and evaluate the quality of data. After the test, raw data were converted to mzXML format with the web-based tool ProteoWizard and then analyzed for peak alignment, retention time correction, and peak area extraction based on XCMS. Metabolite annotation was carried out based on the online human metabolome database (HMDB, http://www.hmdb.ca) using mass-to-charge ratio information and metabolite structures. Metabolite structures were accurately matched using primary and secondary spectrograms (< 25 ppm).

### Statistical analyses

Lipid and metabolite abundance were determined by peak area. Then, data were processed and normalized based on a reference sample (PQN) following the process outlined on the website https://www.metaboanalyst.ca/, which was mainly designed for raw spectra processing and general statistical and functional analysis of targeted metabolomics data [26–28]. The maximum covariance between nontreated samples and MT- or ET-treated samples in lipidomic analysis was determined with partial least squares-discriminant analysis (PLS-DA). The maximum covariance between nontreated samples and MT- or ET-treated samples in metabolomic analysis was determined using orthogonal partial least-squares discriminant analysis (OPLS-DA). The correlation between lipid molecules was analyzed with correlation heatmaps. The content difference of lipids in each sample was indicated with hierarchical clustering analysis. Pathway analysis was carried out with the web-based tool M_ET_PA.

The raw data were logarithmically transformed and tested for normality before the means were compared between different groups. If normality was assumed, Student’s t test was applied. To visualize the differentiation between different groups, PLS-DA and OPLS-DA were performed using MetaboAnalyst 5.0 (http://www.metaboanalyst.ca/). Data are presented as the mean ± SEM. GraphPad Prism (version 8, GraphPad Software, San Diego, CA, USA) was used to perform statistical analyses between the nontreatment and physiotherapy-based treatment groups using Student’s t test (*P* < 0.05).

## Results

### Lipid composition analysis of cNLBP patients before and after manual therapy

**Fig. 1:**
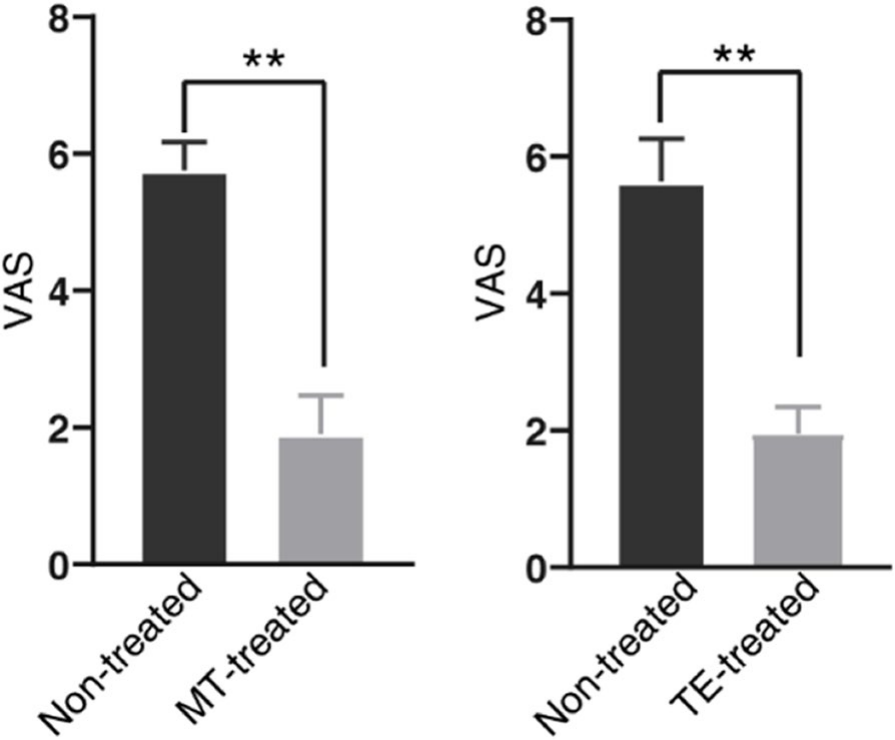
Manual therapy and therapeutic exercise were effective in cNLBP amelioration Seventeen patients were randomly divided into two groups: one group received manual therapy, and the other group received therapeutic exercise. VAS was recorded before and after treatment. Asterisks show a significant difference from patients before treatment using Student’s t tests (**P < 0.01)

We recruited 17 patients with cNLBP whose demographic information is shown in Table 1. The recruited subjects were randomly divided into MT or TE groups, with no significant differences in age, weight, height, BMI, or VAS score between them. We found that MT treatment was effective in alleviating cNLBP (Fig. 1). After treatment, the VAS score decreased in almost the entire MT group (Fig. 1). Serum lipidomics were determined after six MT treatment sessions. Since one participant’s blood sample could not be collected after treatment, there were eight effective participants in the MT group.

**Table 1:** Demographic information among two groups, M ± SEM

	MT	TE	*P*
N (male, count)	8	9	
Age (years)	28.75 ± 7.26	28.11 ± 7.45	not significantly different at baseline
Height (m)	1.68 ± 0.08	1.65 ± 0.05	not significantly different at baseline
Weight (kg)	60.06 ± 8.80	58.00 ± 10.58	not significantly different at baseline
BMI (kg/m^2^)	21.25 ± 2.54	21.17 ± 3.08	not significantly different at baseline
VAS (before treatment)	5.76 ± 1.15	5.63 ± 1.88	not significantly different at baseline
VAS (after treatment)	2.80 ± 1.76	3.47 ± 1.83	not significantly different at baseline

*Abbreviation:* *MT *Manual therapy, *TE* therapeutic exercise, *BMI* Body mass index, *VAS *Visual analog scale (0–10; VAS 0 = no pain; VAS 10 = maximal pain)

**Fig. 2:**
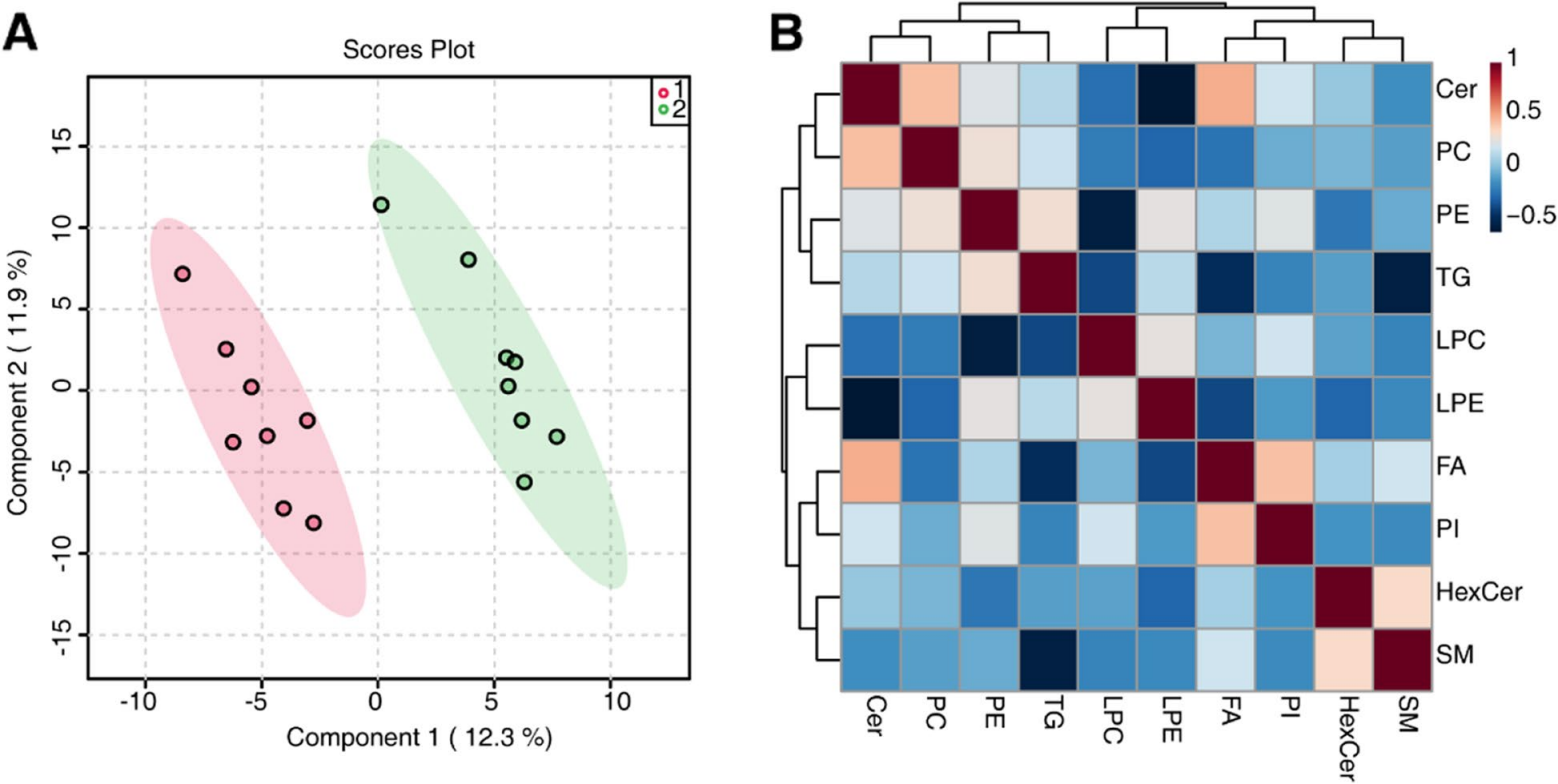
Lipidomic profiles in cNLBP patients before and after treatment with MT. **A** The PLS-DA analysis of cNLBP patients treated with MT versus the control group. “1” represents the nontreated group, and “2” represents the MT-treated group. **B** Correlation analysis of the significantly different lipids. Different colors represent the level of Pearson’s correlation coefficient

**Fig. 3:**
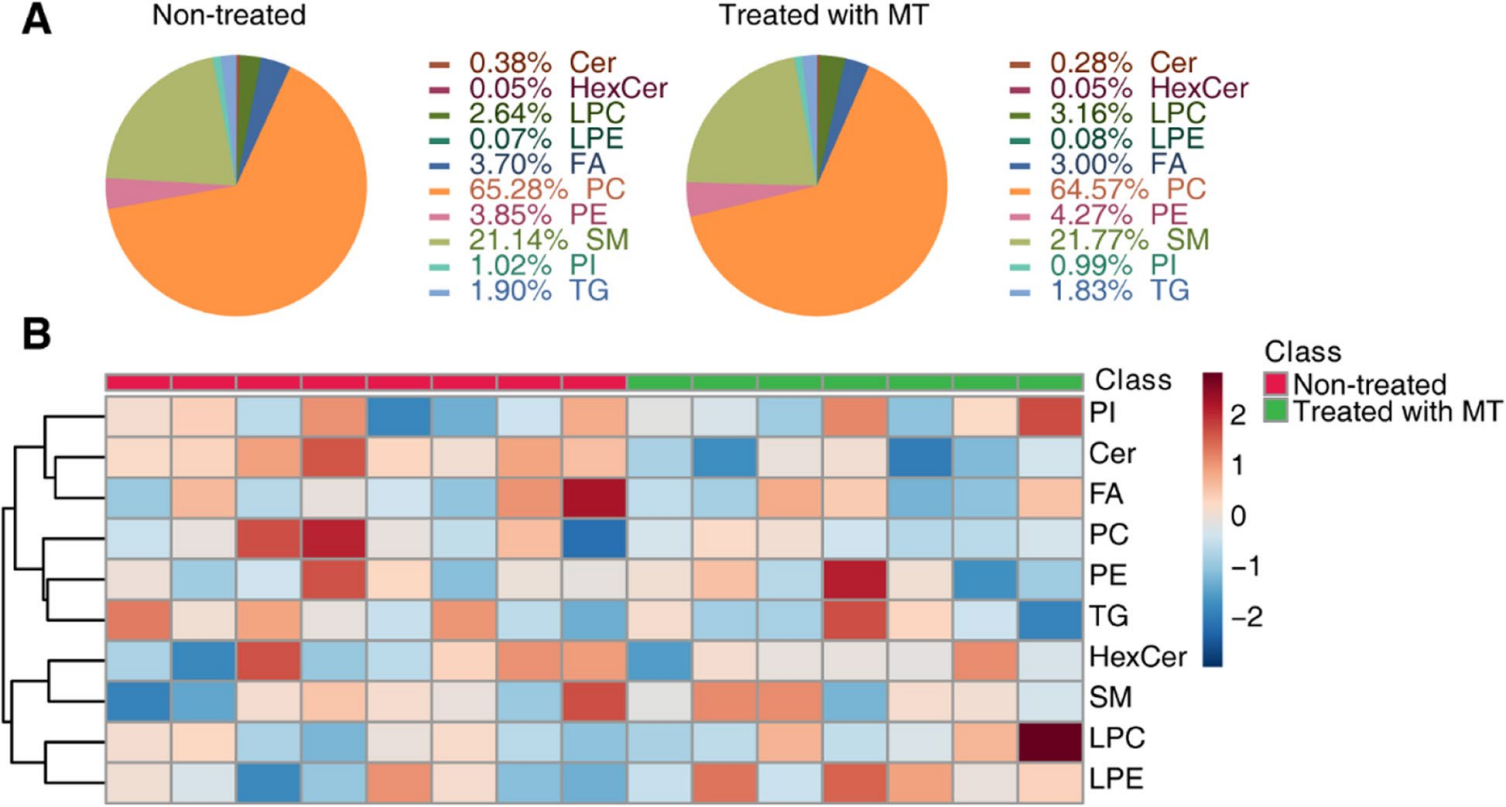
Lipid identification in cNLBP patients before and after treatment with MT. **A** The composition of nontreated samples and MT-treated samples based on the volume of lipids in each lipid category. **B** Hierarchical clustering analysis of the 10 lipids in each sample. For class name, red represents the control group, and green represents the MT-treated group

We completed lipid extraction and performed qualitative analysis. Through lipidomic analysis, we identified 290 lipids, which can be divided into the ten subclasses of phosphatidylcholine (PC), phosphatidylethanolamine (PE), lysophosphatidylcholines (LPC), lysophosphatidylethanolamine (LPE), triacylglycerol (TG), phosphatidylinositol (PI), sphingomyelin (SM), ceramide (Cer), hexosylceramide (HexCer), and fatty acid (FA). As a multivariate statistical analysis, PLS-DA could maximize the distinction and discover different metabolites between groups. We performed PLS-DA analysis with the MetaboAnalyst R software package and found a clear difference in the nontreated group (pink) and MT-treated group (green), suggesting differential lipidomic profiles in cNLBP patients before and after manual therapy (Fig. 2 A). Next, we used Pearson correlation analysis to measure the closeness of different lipids (Fig. 2B). Using volume measurements of lipids, we analyzed the lipidomic composition of cNLBP patients before and after manual therapy and found a decrease in phosphatidylcholine (PC)/phosphatidylethanolamine (PE) molar ratios but an increase in sphingomyelin (SM)/ceramide (Cer) molar ratios in the patients after manual therapy. Meanwhile, there were also decreases in the volumes of fatty acids (FAs) and increases in lysophosphatidylcholine (LPC) and lysophosphatidylethanolamine (LPE) when cNLBP patients were treated with MT (Fig. 3 A). We also generated a heatmap to present the volume of the lipids in each sample (Fig. 3B).

### Lipid composition analysis of cNLBP patients before and after therapeutic exercise (TE)

Therapeutic exercise (TE) is another effective method for improving cNLBP [29]. We found that TE treatment was also effective in alleviating cNLBP (Fig. 1). After treatment, patients’ VAS scores decreased significantly in the TE-treated group (Fig. 1).

**Fig. 4:**
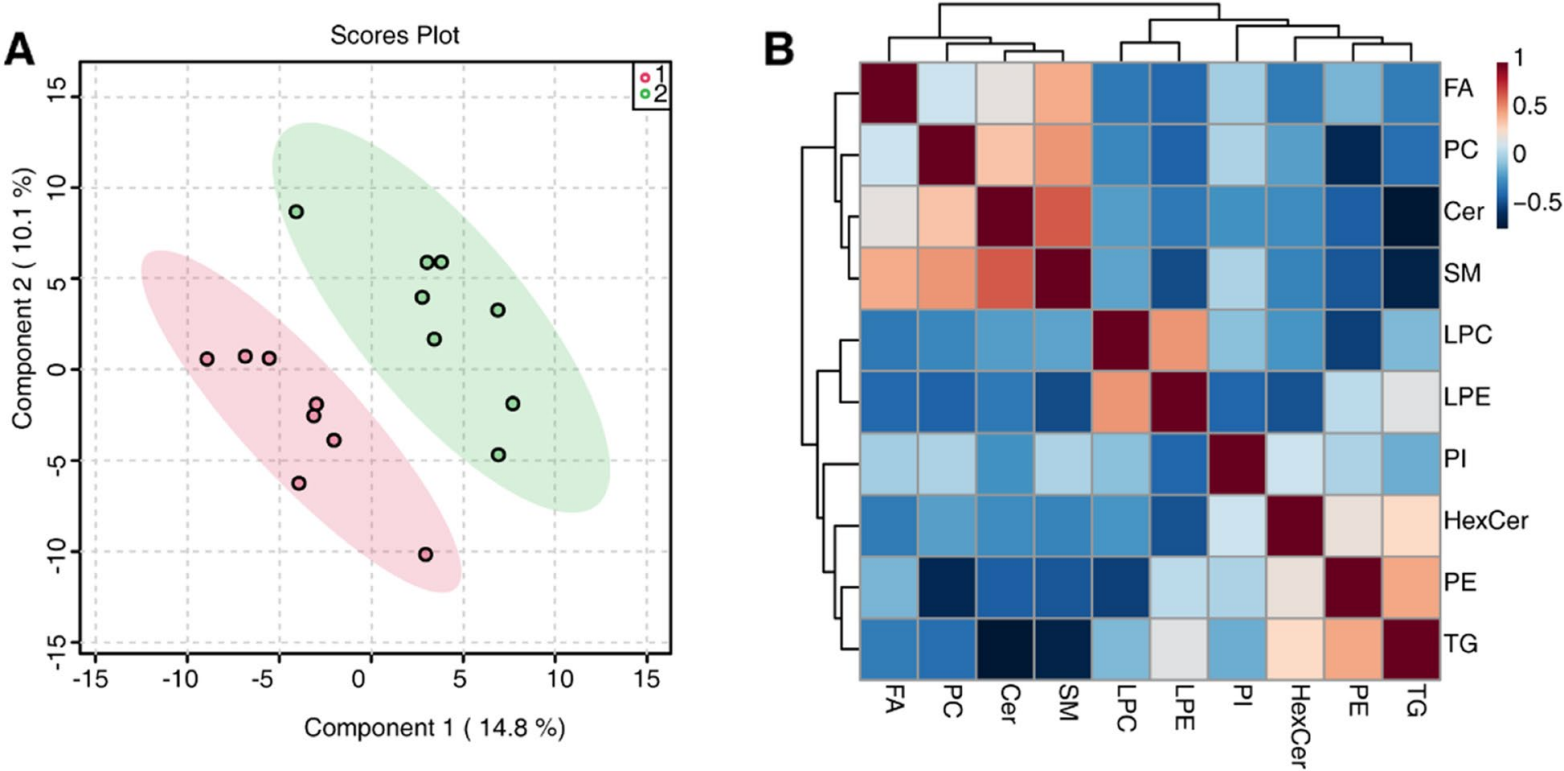
Lipidomic profiles in cNLBP patients before and after treatment with TE. **A** PLS-DA analysis of cNLBP patients treated with TE versus the control group. “1” represents the nontreated group, and “2” represents the TE-treated group. **B** Correlation analysis of the significantly different lipids. Different colors represent the level of Pearson’s correlation coefficient

**Fig. 5:**
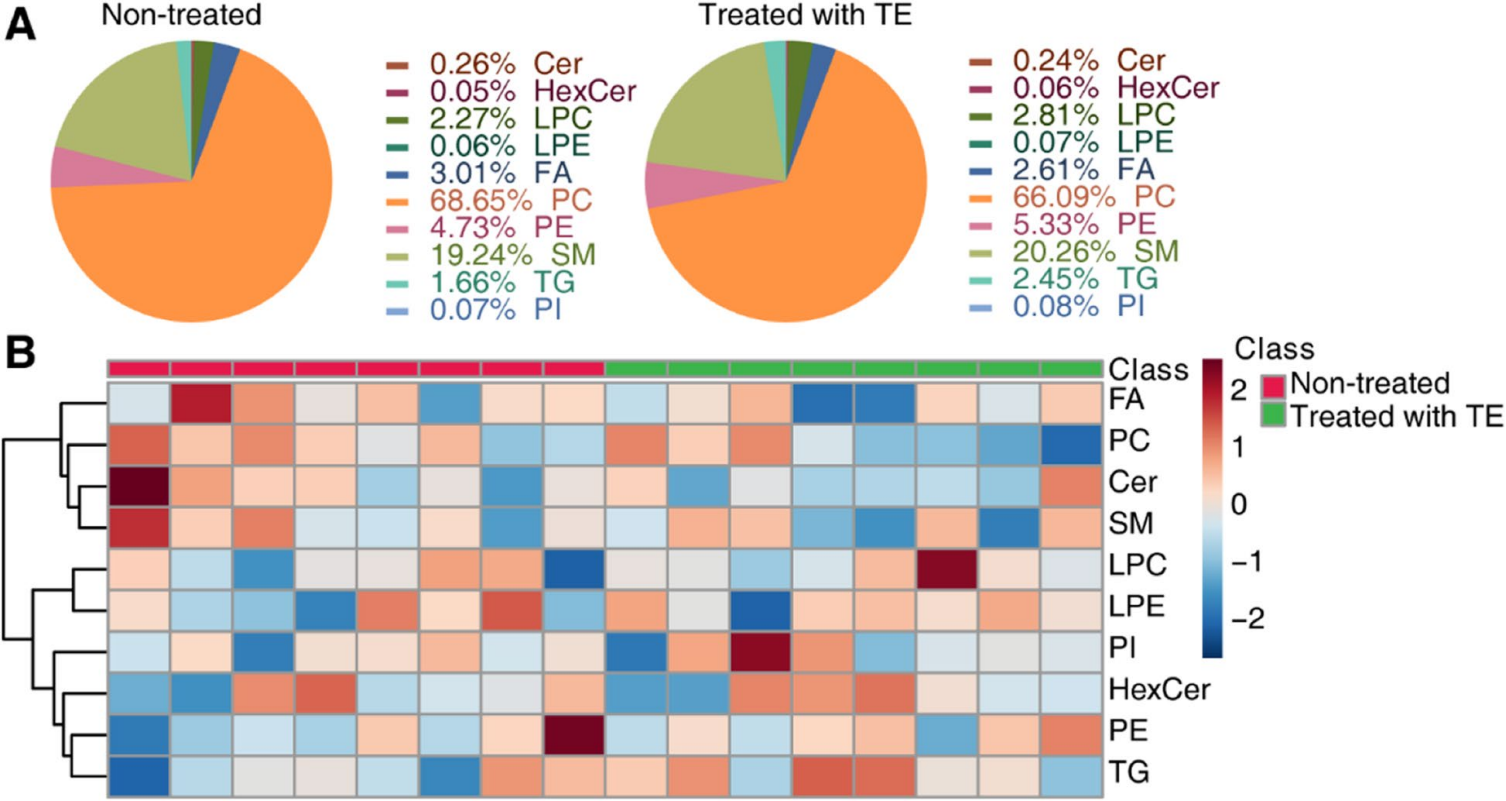
Lipid identification in cNLBP patients before and after treatment with TE. **A** The composition of nontreated samples and TE-treated samples based on the volume of lipids in each lipid category. **B** Hierarchical clustering analysis of the 10 lipids in each sample. For class name, red represents the control group, and green represents the TE-treated group

We performed lipid extraction from cNLBP patients before and after therapeutic exercise and performed qualitative analysis. PLS-DA results indicated a distinct separation between the nontreated group (pink) and the TE-treated group (green) (Fig. 4A). Pearson correlation analysis showed the closeness of different lipids (Fig. 4B). Based on the volume of lipids, PC/PE molar ratios decreased, while SM/Cer molar ratios increased in the patients after therapeutic exercise. The volume of FA also decreased, while the volume of LPC and LPE increased in cNLBP patients after therapeutic exercise, similar to the results of the MT-treated group. Interestingly, the volume of TG (triacylglycerol) increased in the TE-treated group, while it decreased in the MT-treated group (Fig. 5A). A heatmap was produced to indicate the volume of lipids in the nontreated group (red) and the TE-treated group (green) (Fig. 5B).

### Metabolite alterations in cNLBP patients

**Fig. 6:**
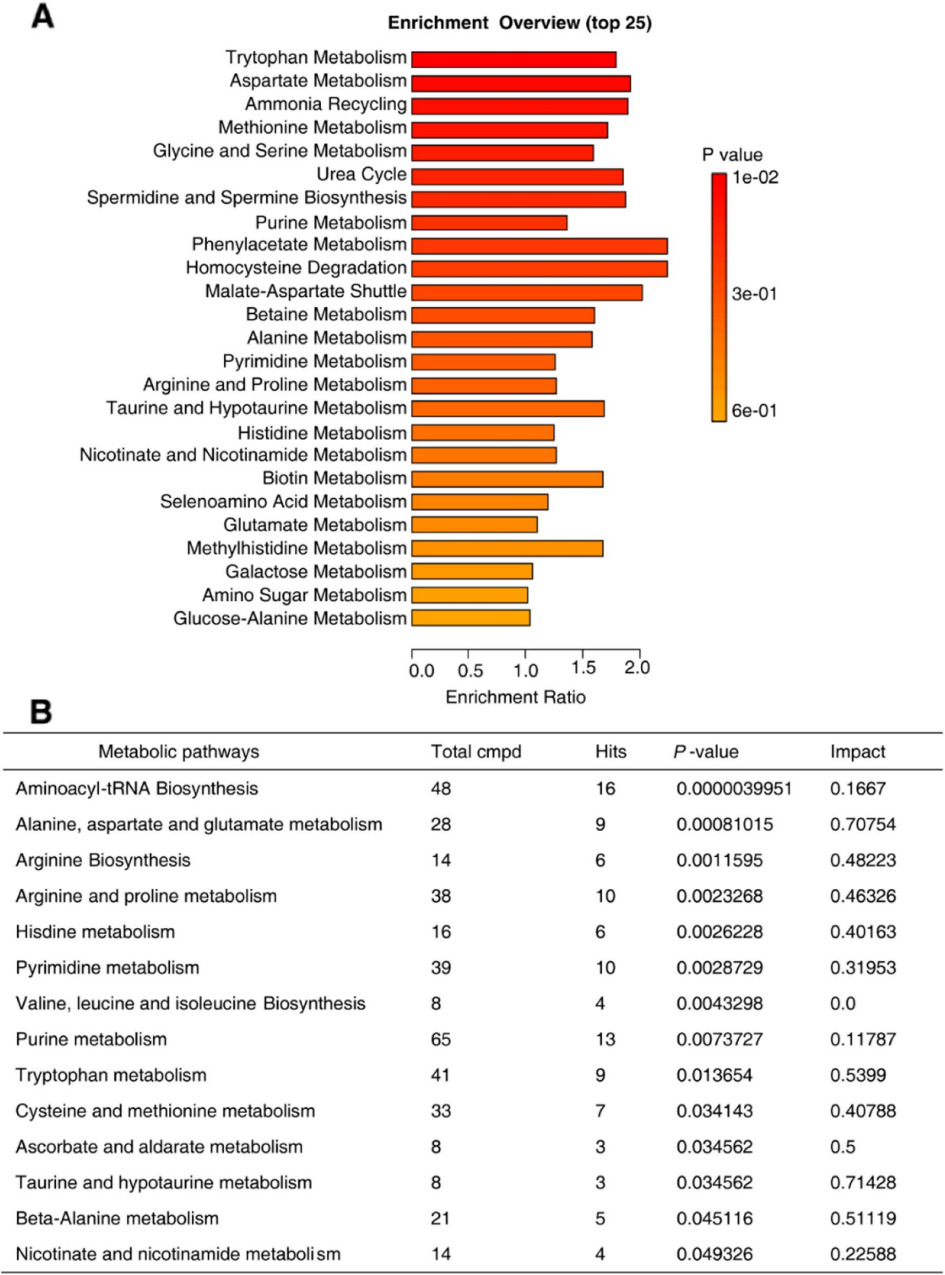
Metabolite alteration in cNLBP patients. **A **Pathway enrichment analysis revealed different metabolic pathways enriched in cNLBP patients (*P* value cutoff ≤ 0.05). **B **The results from the pathway analysis carried out with the web-based tool M_ET_PA using the concentrations of metabolites identified in cNLBP patients. Total cmpd, the total number of compounds in the pathway. Hits are the matched number from the uploaded data. Raw P is the original *P* value. Impact is the pathway impact value calculated from pathway topology analysis

To further identify therapeutic targets for cNLBP physiotherapy-based treatment, we analyzed the metabolome of cNLBP patients before and after treatment. In our sample of patients, the metabolomic analysis annotated and quantified 171 metabolites. Through KEGG-based enrichment analysis, these metabolites were enriched in the metabolism of tryptophan or aspartate, ammonia recycling, the metabolism of methionine or glycine, and serine, among others (Fig. 6A). Combining enrichment and topology analysis, pathway analysis was carried out for all patients. We found a total of 14 pathways that were significantly changed in patients (*P* value < 0.05). These metabolites mainly belonged to aminoacyl-tRNA biosynthesis; arginine biosynthesis; valine, leucine and isoleucine biosynthesis; amino acid metabolism; pyrimidine and purine metabolism; ascorbate and aldarate metabolism; taurine and hypotaurine metabolism; beta-alanine metabolism; and nicotinate and nicotinamide metabolism (Fig. 6B).

### Metabolite profiles of cNLBP patients treated with manual therapy

**Fig. 7:**
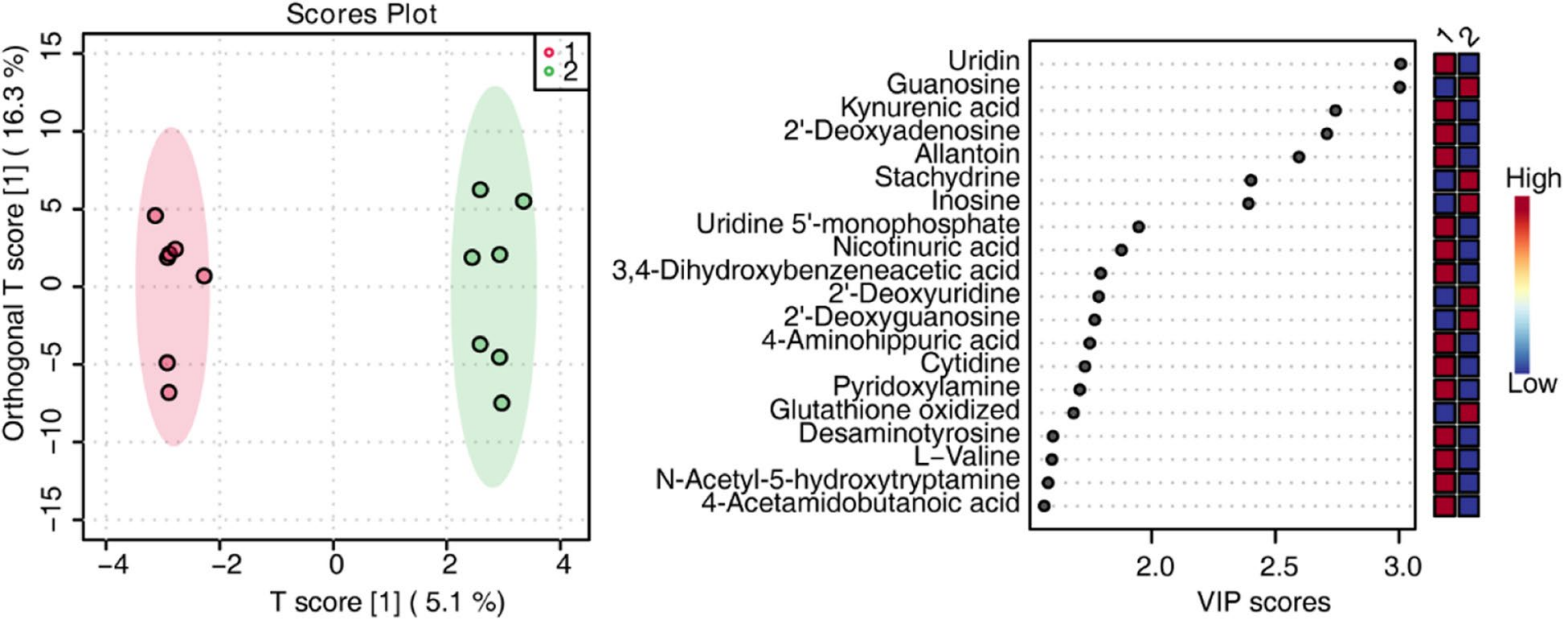
Discrimination through orthogonal PLS-DA of patients before and after manual therapy analyzed based on metabolomics analysis. **A **Orthogonal PLS-DA showing score plots comparing nontreated patients (indicated in the legend as 1) and patients after manual therapy (indicated as 2). **B **Variable importance of projection (VIP) features for the groups from orthogonal PLS-DA analysis

**Fig. 8:**
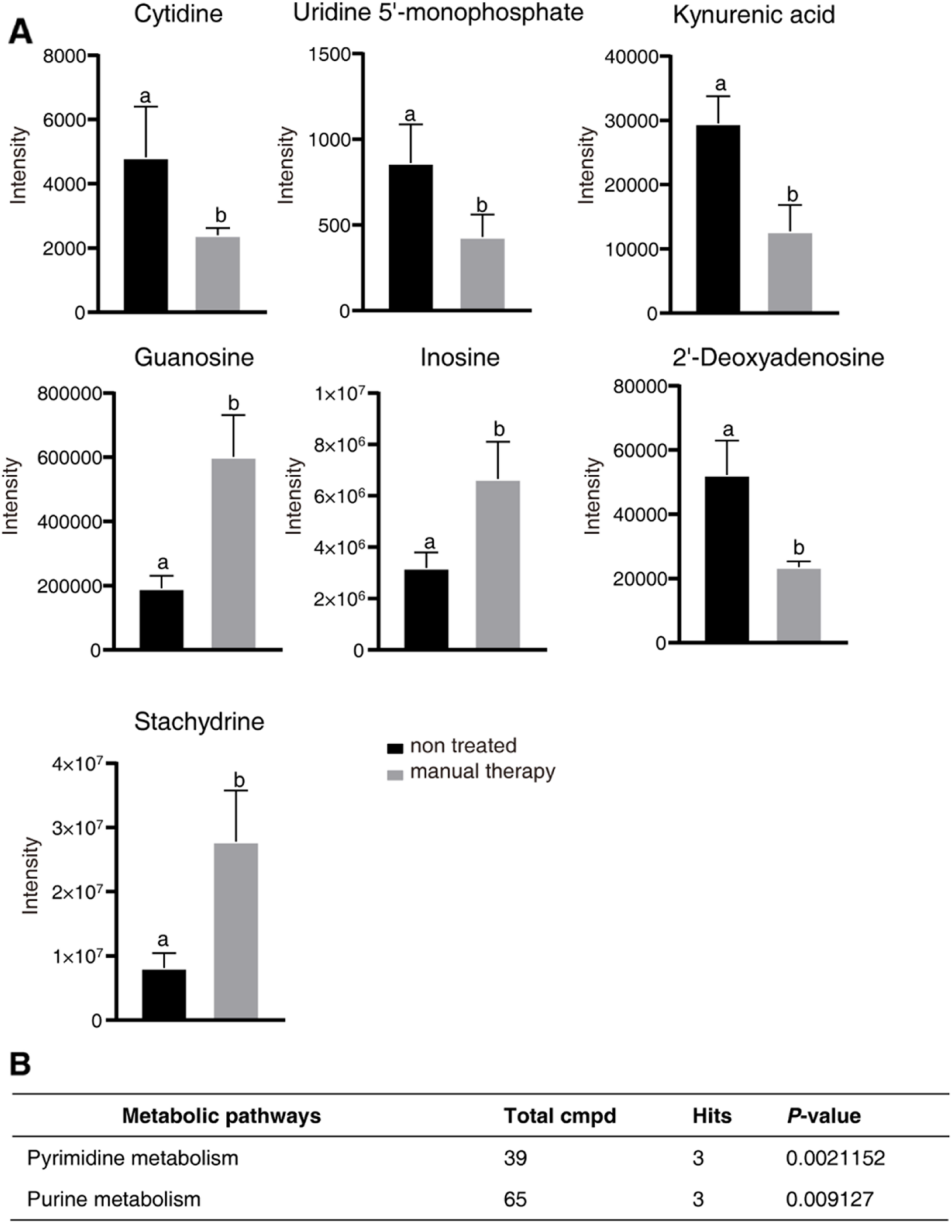
Manual therapy could alter target metabolites in patients with cNLBP. **A **Comparison of the volumes of cytidine, uridine 5’-monophosphate, kynurenic acid, guanosine, inosine, 2’-deoxyadenosine, stachydrine, and N-acetyl-5-hydroxytryptamine in patients treated with and without manual therapy for 2 weeks. Different letters show a significant difference from nontreated patients using Student’s *t* test (*P* < 0.05). **B** Pathway enrichment analysis revealed that pyrimidine metabolism and purine metabolism pathways were enriched in patients treated with manual therapy (*P* value cutoff ≤ 0.05)

Serum metabolome analysis was performed on samples collected after MT treatment. Since two participants’ blood samples could not be collected after treatment, there were seven included participants in the MT group for metabolomes. Orthogonal PLS-DA was performed to demonstrate the suitability of the system (Fig. 7A). The orthogonal PLS-DA score plot revealed good discrimination of the MT treatment group against untreated samples (Fig. 7A). MT-treated and nontreated samples were separated with no outliers (Fig. 7A), demonstrating that our metabolomic analysis could sufficiently reflect the metabolic profile alteration of MT treatment. The VIP scores derived from orthogonal PLS-DA, based on the first 20 metabolites with a VIP score > 1.5, revealed uridine, guanosine, kynurenic acid, 2’-deoxyadenosine, allantoin, stachydrine, inosine, uridine 5’-monophosphate, nicotinuric acid, 3,4-dihydroxybenzeneacetic acid, 2’-deoxyuridine, 2’-deoxyguanosine, 4-aminohippuric acid, cytidine, pyridoxylamine, glutathione oxidized, desaminotyrosine, L − valine, N-acetyl-5-hydroxytryptamine, and 4-acetamidobutanoic acid with the highest VIP scores for MT treatment (Fig. 7B). Finally, we screened out metabolites (fold changes > 2) in the MT treatment group compared with the nontreated group, which were cytidine, uridine 5’-monophosphate, kynurenic acid, guanosine, inosine, 2’-deoxyadenosine, and stachydrine (Fig. 8A). The KEGG-based enrichment analysis revealed that these metabolites were significantly enriched in pyrimidine metabolism and purine metabolism pathways, demonstrating that MT treatment relieves pain by altering the metabolism of these two pathways (Fig. 8B).

### Metabolite profiles of cNLBP patients treated with therapeutic exercise

**Fig. 9:**
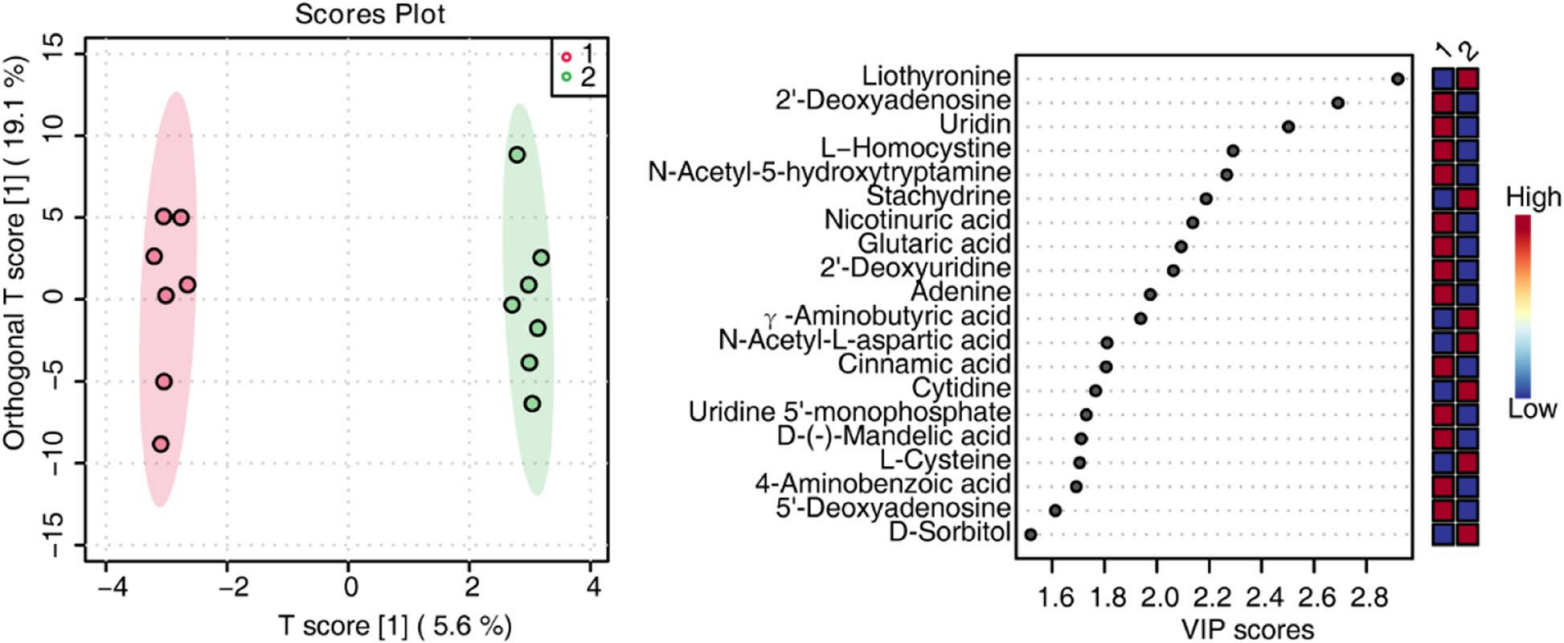
Discrimination through orthogonal PLS-DA of patients before and after therapeutic exercise examined by metabolomic analysis. **A **Orthogonal PLS-DA showing score plots comparing nontreated patients (indicated in the legend with 1) and patients after therapeutic exercise (indicated with 2). **B** Variable importance of projection (VIP) features for the groups from orthogonal PLS-DA analysis

We also performed metabolite identification in the serum metabolomes pooled from cNLBP patients treated with TE. Since one participant’s blood sample could not be collected after treatment, there were seven included participants in the TE group for metabolomes. Orthogonal PLS-DA was performed on untreated samples and TE treatment samples. The orthogonal PLS-DA score plot revealed good discrimination between the TE treatment group and the nontreated samples (Fig. 9A). The VIP score > 1.5 derived from orthogonal PLS-DA, based on the first 20 metabolites, revealed liothyronine, 2’-deoxyadenosine, uridine, L − homocystine, N-acetyl-5-hydroxytryptamine, stachydrine, γ-aminobutyric acid, nicotinuric acid, glutaric acid, 2’-deoxyuridine, adenine, N-acetyl-L-aspartic acid, cinnamic acid, cytidine, uridine 5’-monophosphate, D-(-)-mandelic acid, L-cysteine, 4-aminobenzoic acid, 5’-deoxyadenosine, and D-sorbitol with the highest VIP scores for TE treatment (Fig. 9B).

**Fig. 10:**
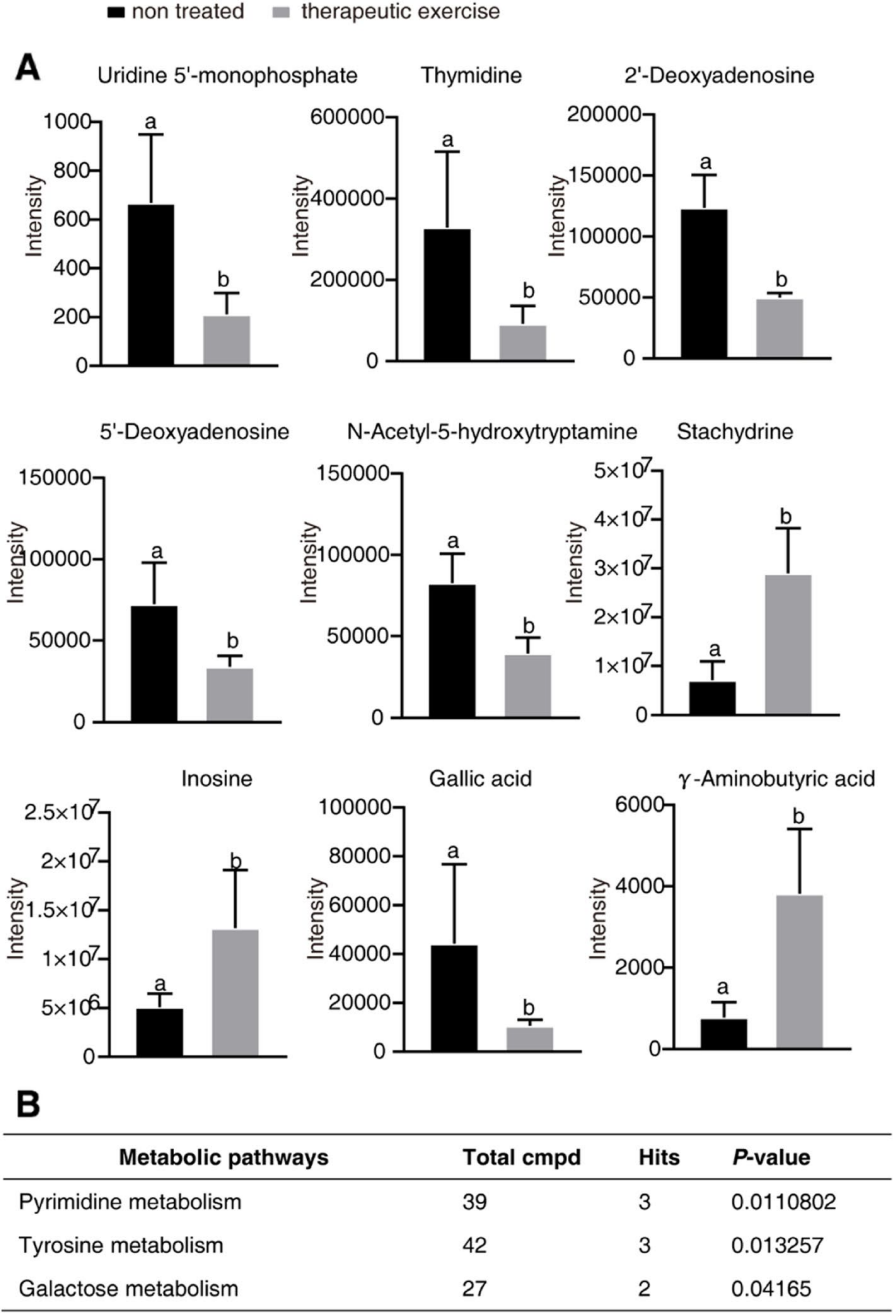
Therapeutic exercise could alter target metabolites in patients with cNLBP. **A **Comparison of the volumes of uridine 5’-monophosphate, thymidine, 2’-deoxyadenosine, 5’-deoxyadenosine, N-acetyl-5-hydroxytryptamine, stachydrine, inosine, gallic acid, and γ-aminobutyric acid in patients treated with therapeutic exercise for two weeks or without therapeutic treatment. Different letters show a significant difference from nontreated patients using Student’s t test (*P* < 0.05). **B** Pathway enrichment analysis revealed that pyrimidine metabolism, tyrosine metabolism, and galactose metabolism were enriched in patients treated with therapeutic exercise (*P* value cutoff ≤ 0.05)

Finally, nine metabolites with fold changes > 2 in the TE treatment group were found, including uridine 5’-monophosphate, thymidine, 2’-deoxyadenosine, 5’-deoxyadenosine, N-acetyl-5-hydroxytryptamine, stachydrine, inosine, gallic acid, and γ-aminobutyric acid (Fig. 10A). The KEGG-based enrichment analysis revealed that these nine metabolites were significantly enriched in pyrimidine metabolism, tyrosine metabolism, and galactose metabolism pathways, demonstrating that TE treatment relieves pain by altering the metabolism of these three pathways (Fig. 10B).

## Discussion

Lipids can act as bioactive compounds that play critical roles in signal transduction. The balance of cellular PC/PE molar ratios is crucial to maintain cell survival and participates in the regulation of many diseases. However, PC/PE molar ratios associated with physiotherapy with cNLBP have not been studied. In this study, we found that PC/PE molar ratios decreased in cNLBP patients either treated with MT or treated with TE when compared with control groups, suggesting that PC/PE molar ratios are involved in cNLBP physiotherapy-based treatment. We still do not know the exact reason why decreased PC/PE molar ratios induced by MT or TE can cause cNLBP relief. However, we believe that the most likely explanation is that decreased PC/PE can alter the properties of membranes and inhibit TNFa-induced inflammatory responses significantly, which is an important inducer of sensory nerve growth [30, 31]. Studies have also shown that the growth of sensory nerves into the inner layer of IVDs (intervertebral discs, IVDs) is a potential factor in low back pain [32, 33].

Sphingolipids are another kind of bioactive lipid that can be used as powerful signaling molecules, and dysregulation of sphingolipid metabolism changes is known to have a significant impact on signal transduction [34]. Sphingomyelin (SM) and ceramide (Cer) are the most enriched classes of sphingolipids, and the balance between SM and Cer is associated with human disease. For example, SM/Cer imbalance can promote lipid dysregulation and apoptosis [35]. Studies have shown that altered sphingolipid metabolism causes neuropathic pain in humans [36]. *N,N*-dimethylsphingosine induces mechanical hypersensitivity, *and* the SM/Cer ratio is altered in rats with neuropathic pain [37]. There has, however, been no investigation examining whether the changes in the SM/Cer ratio were related to the physiotherapy of cNLBP. In our study, we found that the SM/Cer ratio increased in cNLBP patients treated with MT or TE compared with control groups, suggesting that SM/Cer ratio alteration is involved in cNLBP physiotherapy-based treatment. However, thus far, there has been no study on the mechanism of the SM/Cer ratio in cNLBP. SM can be hydrolyzed to produce biologically active molecules, such as ceramide and sphingosine, which can be used as potent inhibitors of protein kinase C (PKC) [38]. Therefore, SM/Cer ratio alterations can control many signaling pathways related to inflammation through PKC to relieve low back pain, since inflammation is the primary source of low back pain [28, 39]. In addition, SM/Cer ratio alterations can decrease chronic inflammatory responses through ER stress [40].

We further performed metabolome analysis to identify the underlying mechanisms in cNLBP physiotherapy-based treatment through MT or TE. We found that pyrimidine metabolism and purine metabolism pathways related to MT caused cNLBP amelioration, while pyrimidine metabolism, tyrosine metabolism, and galactose metabolism pathways were responsible for TE-generated cNLBP amelioration. There is literature demonstrating that pyrimidines and purine have widespread functions in responding to pain therapeutics [41, 42]. For example, the nucleotides cytidine and uridine are helpful for dealing with low back pain [43]. The amount of tyramine sulphate was significantly lower in pain patients than in control patients [41]. Purine antagonists can reduce chronic pain and inflammatory pain. Adenosine and its analogs have the ability to suppress nociception by activating adenosine receptors [44]. In addition to the pyrimidine pathway, tyrosine metabolism is also associated with pain [45]. In headache patients, tyrosine metabolism levels are abnormal [46]. Tyrosine can be hydrolyzed to DOPA, dopamine (DA), and noradrenaline (NE), which govern pain and vegetative functions [47]. Galactose was not only used as a primary source of energy but also considered a candidate for pharmacological applications [48].

In comparing the lipidomic and metabolomic profiles of patients with cNLBP before and after treatment, we found that alterations in the PC/PE ratio, SM/Cer ratio, and target metabolites may be the cause of cNLBP amelioration by MT or TE. However, the relationship between lipids and target metabolites is still unclear. We still do not know whether lipid alteration affects metabolite volume or whether metabolite volume affects lipid alteration. Many studies have demonstrated that lipids can affect gene expression, which can then alter the level of metabolites [49, 50]. For example, S1P can inhibit the activity of histone deacetylases by binding with HDAC1 and HDAC2 specifically to the epigenetic regulation of gene expression [49]. Lipids can also directly affect the activity of protein kinase C, which is an important downstream target of Cer. They can also modulate pyrimidine biosynthesis [50]. In turn, metabolite alteration can also affect lipid metabolism. For example, prenyloxycoumarin is a secondary metabolite and can be used as a modulator of lipid metabolism [51]. Very-low-density lipoproteins (VLDL) are a risk factor for modic changes. These changes result in low back pain (LBP), and receptors can enhance lipid metabolism and promote the expression of interleukin-33 (IL-33) [9, 52]. More studies are needed, however, to investigate the relationship between lipid metabolism and metabolite metabolism in the process of MT or TE in reducing cNLBP. Our study identified the target lipids and metabolites involved in the improvement of cNLBP treated with MT or TE, which has expanded our knowledge of cNLBP physiotherapy-based treatment.

### Study strengths and limitations

The greatest strength of this study is to reveal the possible mechanism of promoting cNLBP amelioration through MT or TE treatment from the perspective of lipidomics and metabolomics in cNLBP patients. However, the experiment only involved with alterations in lipids and metabolites, and the deeper mechanisms of these lipids and metabolites affecting cNLBP physiotherapy-based treatment are uncertain. Therefore, more evidences are needed to explore.

## Conclusions and clinical perspective

MT or TE treatment were effective strategies in alleviating cNLBP. The possible mechanism is that MT or TE treatment was able to cause alterations in the lipidomics and metabolomics in cNLBP patients. This study was the first to elucidate cNLBP physiotherapy-based treatment was associated with specific lipids and metabolites. These results indicate that physiotherapy or agents targeting these lipids and metabolites alteration might be useful for treatment of cNLBP.

## Data Availability

All data in this study can be obtained from the corresponding author upon request.
